# Cyclosporine therapy could be considered for membranoproliferative glomerulonephritis with immunoglobulin A deposits: a case report

**DOI:** 10.1186/s12882-022-02983-5

**Published:** 2022-11-07

**Authors:** Yuko Hidaka, Hiroshi Tamura, Keishiro Furuie, Shohei Kuraoka, Hiroko Nagata, Hitoshi Nakazato

**Affiliations:** Department of Pediatrics, Faculty of Life Sciences, Kumamoto University, 1-1-1 Honjo, Kumamoto, 860-8556 Japan

**Keywords:** Membranoproliferative glomerulonephritis, IgA2 dominant deposits, Cyclosporine

## Abstract

**Background:**

Membranoproliferative glomerulonephritis (MPGN), a rare glomerulonephritis that causes nephrotic syndrome in children, is often difficult to treat. Typical immunofluorescence findings include strong C3 staining in a granular pattern along the glomerular capillary wall and negative IgA staining. IgA-dominant MPGN without hypocomplementemia has been reported. Herein, we report a rare case of MPGN with hypocomplementemia and predominant IgA subclass 2 deposits.

**Case presentation:**

An 11-year-old girl showed proteinuria on a school urinalysis screening and presented with upper eyelid edema. The urinalysis showed elevated urinary protein levels and hematuria. Laboratory examinations revealed the following: serum albumin, 1.3 g/dL; serum creatinine, 0.54 mg/dL; and C3c, 67 mg/dL (normal range: 73–138 mg/dL). The physical and laboratory findings did not suggest autoimmune diseases. A renal biopsy was then performed. Specimen examination under a light microscope showed mesangial cell proliferation, increased mesangial matrix with lobulation, and some double contours of the glomerular basement membrane in almost all glomeruli, which are characteristic findings of MPGN. Immunofluorescent studies showed IgA deposits not only in the mesangial regions but also along the capillary walls, which were more strongly stained than C3. IgA subclass staining showed a stronger immunoreactivity for IgA2 than IgA1. Electron microscopic studies showed electron-dense deposits in the subendothelial, subepithelial, and paramesangial regions. Based on these findings, the patient was diagnosed with IgA-dominant MPGN. Accordingly, she was treated with three courses of methylprednisolone pulse therapy (MPT), followed by prednisolone, mizoribine, and lisinopril. Although hypocomplementemia improved after three courses of MPT, nephrotic-range proteinuria and hypoalbuminemia remained; therefore, two courses of MPT were additionally administered, and the immunosuppressant was changed from mizoribine to cyclosporine (CsA). Finally, the urinary protein level decreased, and a subsequent renal biopsy, two years later, showed improvement in the lesions.

**Conclusions:**

We report an atypical case of MPGN with IgA2 dominant deposits along the glomerular capillary wall and in the mesangial region. The case was refractory to standard therapy but sensitive to CsA, which resulted in remission. Our findings suggest that CsA may be useful as an immunosuppressant to treat refractory MPGN.

## Background

Most childhood nephrotic syndromes are minimal change diseases, and occurrence of nephritic nephrosis with hematuria is rare. In IgA nephropathy, the most common glomerular disease in children and adolescents, most IgA deposits are mainly detected in the mesangial region, and only some are found in the glomerular capillary wall [1]. IgA deposits may also be observed in other diseases, but IgA deposits along the glomerular capillary wall is not common, except for in some collagen diseases. In this case study, using histopathology of renal biopsy specimens, we diagnosed a case of membranoproliferative glomerulonephritis (MPGN) with IgA deposits along the glomerular capillary wall and in the mesangial region in a child with nephrotic syndrome who developed hematuria. No concomitant disease was observed, and IgA subclass 2 was dominant in the deposited IgA molecules. This patient was diagnosed as a case of primary renal disease. Herein, we report the clinicopathologic features and treatment of this case.

## Case presentation

An 11-year-old girl showed proteinuria on a school urinalysis screening. She was taken to a hospital for investigation of upper eyelid edema. She was born at 26 weeks of gestation, with a birth weight of 526 g (standard deviation [SD], -2.7) and birth height of 30 cm (SD, -1.3), and she started growth hormone treatment at the age of 4 years and 5 months for her short stature. There was no medical or family history of kidney disease. She had no infection in the last few months, and her vital signs were normal. Her height was 137 cm, and her weight was 37 kg, which was 7 kg heavier than usual. Physical examination showed that she had marked generalized edema. She had no cutaneous purpura, abdominal pain, or joint pain, suggesting that she did not have IgA vasculitis. Urinalysis and urine chemistry results were as follows: urinary protein, 2165 mg/dL; urinary protein-creatinine ratio, 22 g/gCr; urinary red blood cell count, > 100 /high power field; and urinary β2-microglobulin, 8579 μg/L. The findings of laboratory examinations were as follows: normal complete blood cell count; serum total protein, 3.7 g/dL; serum albumin, 1.3 g/dL; aspartate aminotransferase, 20 IU/L; alanine aminotransferase, 14 IU/L; lactate dehydrogenase, 159 IU/L; alkaline phosphatase, 383 IU/L; total bile acid, 1.1 μmol/L; ammonia, 33 μg/dL; total cholesterol, 666 mg/dL; triglyceride, 268 mg/dL; blood urea nitrogen, 12.9 mg/dL; serum creatinine, 0.54 mg/dL; sodium, 135 mEq/L; potassium, 4.5 mEq/L; chloride, 107 mEq/L; calcium, 7.4 mg/dL; and C-reactive protein, 0.02 mg/dL. Immunological examination revealed serum IgG of 122 mg/dL, serum IgA of 82 mg/dL, C3c of 67 mg/dL (normal range: 73–138 mg/dL), C4 of 14.1 mg/dL (normal range: 11–31 mg/dL), CH50 of 28 U/mL, and antistreptolysin O of 44 IU/mL. Antinuclear, myeloperoxidase, and proteinase 3 antineutrophil cytoplasmic antibodies were negative. Screening for infectious diseases were negative for parvovirus B19 IgM and mycoplasma antibodies. Her physical and laboratory findings suggested no autoimmune diseases or infections. Hepatitis B surface antigen and hepatitis C antibody test results were also negative. Liver disease-related IgA nephropathy was ruled out because ultrasonography and computed tomography revealed ascites in the absence of portosystemic shunt and cryoglobulinemic glomerulonephritis was ruled out because blood tests were negative for cryoglobulin. Based on these results, nephrotic nephritis with hypocomplementemia was suspected, and a renal biopsy was thus performed.

**Fig. 1:**
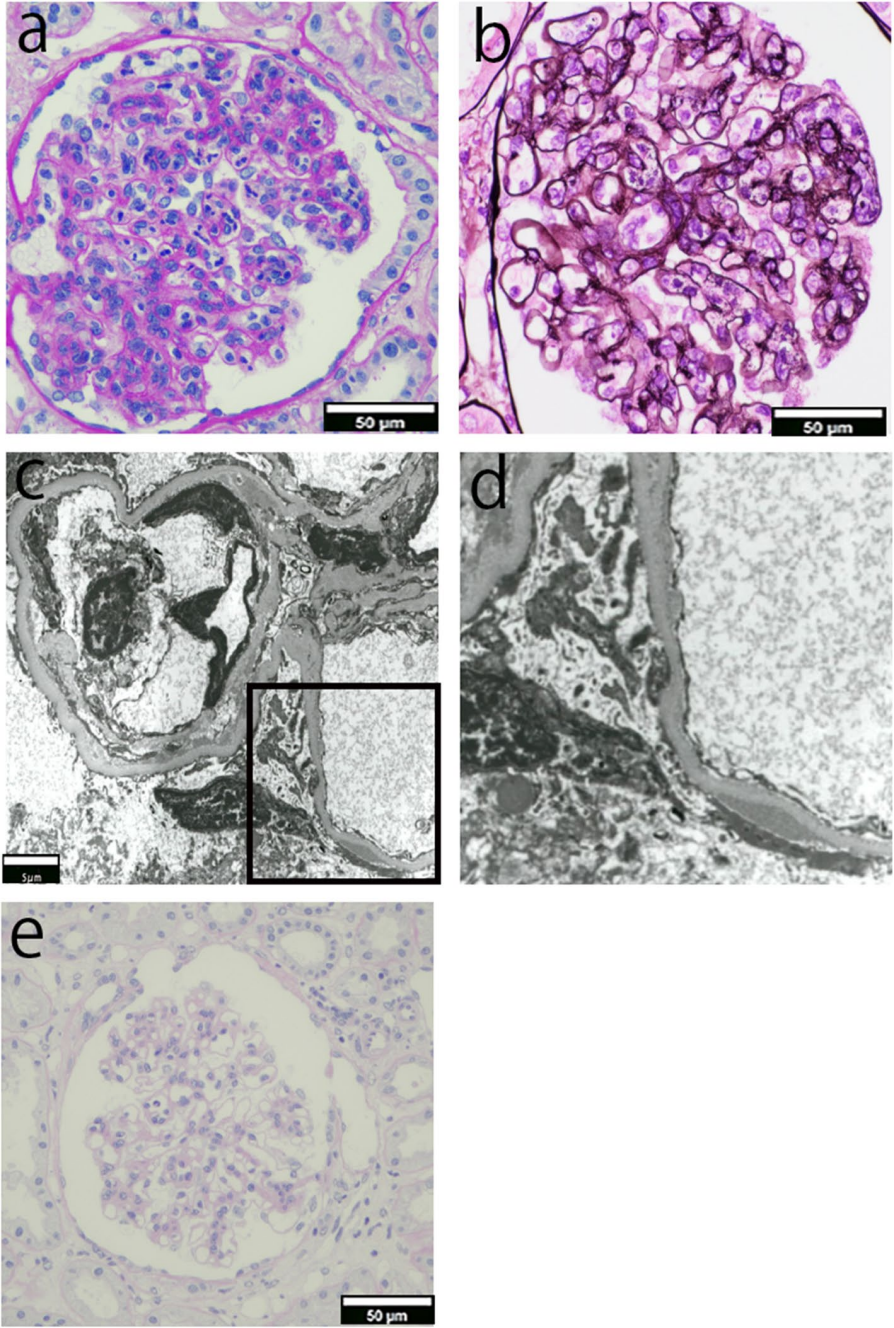
Pathological results of the first and second kidney biopsies. Light and electron microscopy Nikon ECLIPSE Ts2R and NIS Elements (Nikon Corporation, Japan) is used to capture the images. The images were obtained with eyepiece at 10X magnification and objective at 20X. The brightness and contrast were adjusted by using Phtoshop (adobe, Japan). No other downstream processing or averaging were applied to the images to enhance the resolution. **a** Periodic acid Schiff (PAS) staining of the first kidney biopsy showed mesangial cell proliferation and increased mesangial matrix with lobulation. Endocapillary proliferation in the glomeruli and a few neutrophil infiltrations in the capillaries of the glomeruli were observed (Magnification: × 200). **b** Periodic Acid-Methenamine-Silver staining of the first kidney biopsy showed some double contours of the glomerular basement membrane (Magnification: × 200). **c** Electron microscopy analysis showed electron-dense deposits in the subendothelial, subepithelial, and paramesangial regions (black box; Magnification: × 3000). **d** Enlarged views of marked regions (black box) in corresponding Fig. 1-c image. (Magnification: × 3000) Electron microscopy also showed that the subepithelial deposits were not hump-like and podocytes were partially sparse. **e** PAS staining of the second biopsy two years later showed mild proliferation of mesangial cells (Magnification: × 200)

**Fig. 2:**
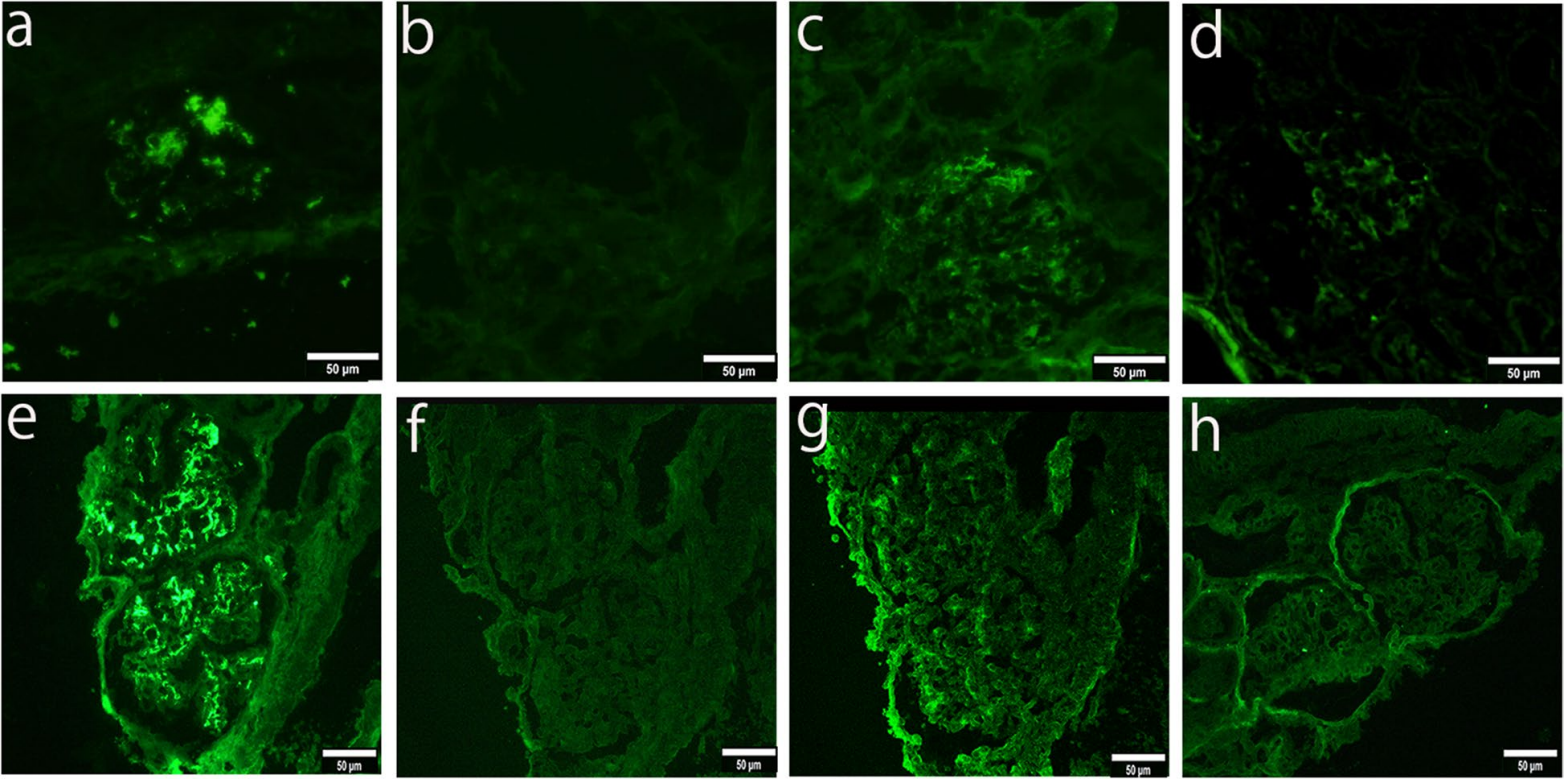
Immunofluorescent studies of the first and second renal biopsies. Fluorescence microscope microscopy Nikon ECLIPSE Ts2R and NIS Elements (Nikon Corporation, Japan) is used to capture the images. The images were obtained with eyepiece at 10X magnification and objective at 20X. The brightness and contrast were adjusted by using Phtoshop (adobe, Japan). No other downstream processing or averaging were applied to the images to enhance the resolution. On the first biopsy (**a**-**d**), positive deposits of IgA (**a**) and C3 (**d**) were dominantly detected in the mesangial regions, and were also slightly detected along the capillary walls. In addition, IgA deposits were stronger than those of C3. IgA2 (**c**) staining was stronger than IgA1 (**b**) staining. On the second biopsy (**e**–**h**), positive deposits of IgA (**e**) were detected mainly in the mesangial regions and also slightly along the capillary walls. C3 (**h**) deposits were not detected. IgA1 (**f**) deposits were not found, and IgA2 (**g**) deposits were found in the mesangial region. (Magnification: × 200, a-h)

**Fig. 3:**
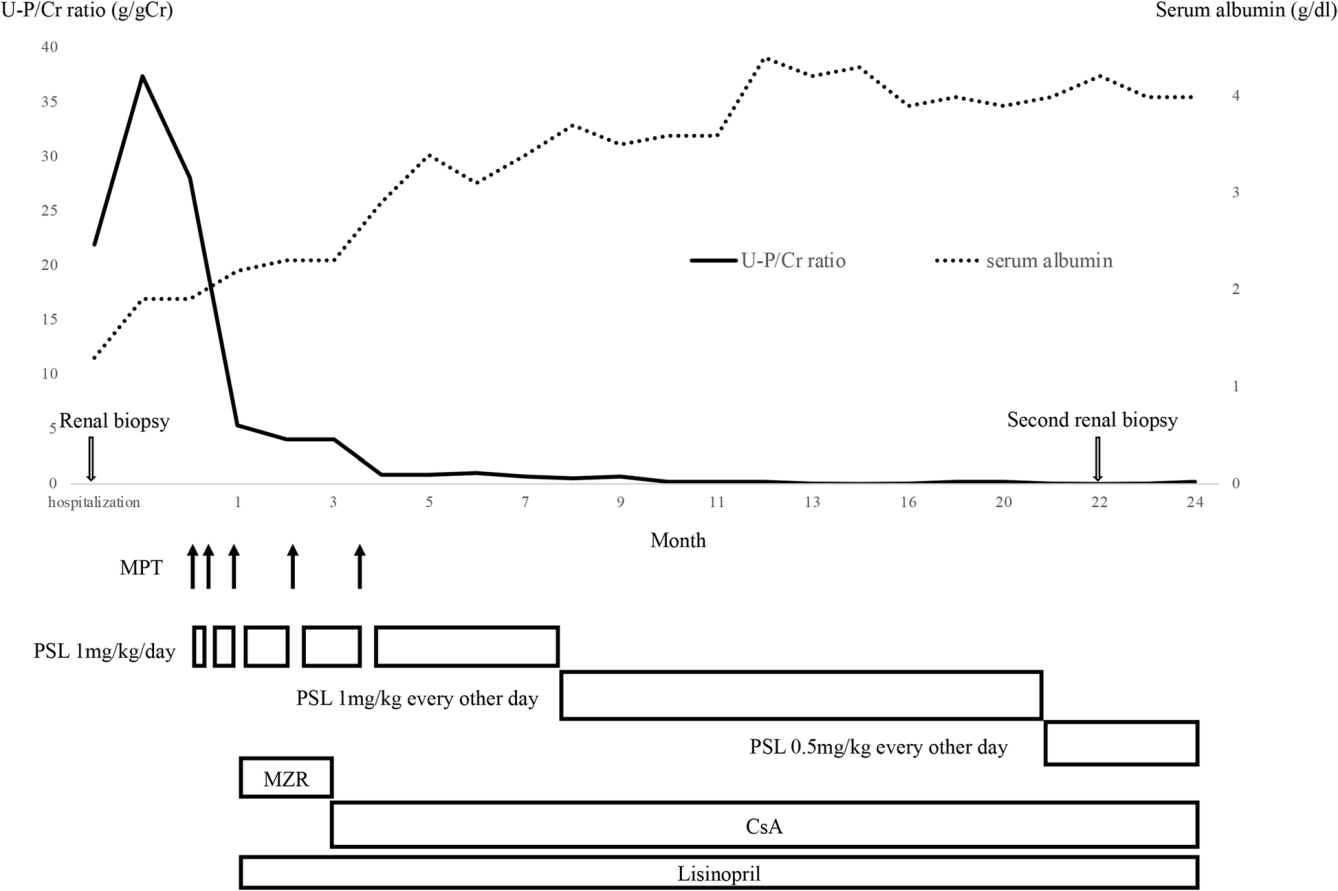
Urinary protein-creatinine ratio (U-P/Cr ratio) and serum albumin levels at 24 months from the start of treatment

Light microscopic studies showed mesangial cell proliferation and increased mesangial matrix with lobulation in almost all glomeruli. Endocapillary proliferation in the glomeruli and a few neutrophil infiltrations in the capillaries of the glomeruli were observed (Fig. 1a). Additionally, periodic acid-methenamine-silver staining showed some double contours of the glomerular basement membrane (Fig. 1b); these are all characteristic findings of MPGN. The size of the glomeruli was within the normal range, and there were 2.6 glomeruli per square millimeter. The glomerular density of 13 minimal change nephrotic syndrome patients (mean 11.8 ± 3.6 years) with normal birth weight was 3.6 ± 1.1 / per square millimeter [2], indicating that the glomerular density of this patient was within the normal range or slightly lower. Immunofluorescent studies showed that deposits of IgA and C3 were dominantly detected in the mesangial regions, and were also slightly detected along the capillary walls. In addition, IgA deposits were stronger than those of C3 (Fig. 2a, d). IgA subclass staining revealed stronger staining for IgA2 stains than for IgA1 stains (Fig. 2b-c). Electron microscopic studies showed electron-dense deposits in the subendothelial, subepithelial, and paramesangial regions. Mesangial cell interposition was observed (Fig. 1c). The subepithelial deposits were not hump-like and podocytes were slightly sparse. No disease-specific glomerular deposit structures such as renal amyloidosis or monoclonal immunoglobulin deposition disease were observed on EM (Fig. 1c-d). Based on these aforementioned findings, we diagnosed this girl with IgA-dominant MPGN. Hypoalbuminemia was treated by intravenous infusion of albumin, and furosemide was also administered. After the biopsy, the patient was treated with three courses of methylprednisolone pulse therapy (MPT; one course; 30 mg/kg of methylprednisolone for three consecutive days), followed by oral prednisolone (1 mg/kg/day) and mizoribine (MZR). Lisinopril was used to treat hypertension and proteinuria. Although hypocomplementemia improved after the three courses of MPT, nephrotic-range proteinuria and hypoalbuminemia remained; therefore, two courses of MPT were additionally administered, and the immunosuppressant was changed from MZR to cyclosporine (CsA). The dose of CsA was initially started at 2 mg/kg/day. The CsA blood level target for single-point concentration 2 h post-dose was set at 600–700 ng/mL for approximately 2 months, after which the dosage was adjusted to 450–550 ng/mL. Subsequently, the urinary protein-creatinine ratio decreased from 4 to 0.8 g/gCr, and serum albumin tended to increase. She was discharged on the 155^th^ day of hospitalization. The dose of prednisolone was changed every other day and gradually decreased. Decreased urinary protein was detected 13 months after the treatment (Fig. 3). A second renal biopsy was performed 22 months after the start of treatment, and improvement in glomerular lesions was found (Fig. 1e). Cyclosporine nephropathy was not observed. On the second biopsy, positive deposits of IgA were detected mainly in the mesangial regions and also slightly along the capillary walls. Deposits of C3 and IgA1 were not detected, but very slight deposits of IgA2 were present in the mesangial regions and along the capillary walls. (Fig. 2e-h). No deterioration of kidney function was observed after almost 2 years of treatment. The levels of C3 improved after three courses of methylprednisolone pulse therapy, and has remained normal since then. Posterior subcapsular cataract was considered as an adverse effect of steroids because no deterioration in visual acuity was observed during the follow-up period.

## Discussion and conclusions

Accumulating studies reveal normal complement levels in cases of pediatric IgA nephropathy with MPGN-like patterns [3, 4]. For example, Andeen et al. found that among 15 IgA-dominant MPGN patients without liver disease, 11 (73%) had nephrotic-level proteinuria, and the complement level was normal [5]. Here, we report a rare case of IgA-dominant MPGN with hypocomplementemia.

In IgA nephropathy, the alternative pathway is activated along the capillary wall and in the mesangium because of C3 deposits [6]; IgA1 deposits in the mesangium activates the alternative pathway, and IgA2 deposits activates the lectin pathway [7]. In our case, IgA2 staining was stronger than IgA1 staining, suggesting predominant IgA2 deposits. No symptoms and test results suggested infectious diseases before the onset of MPGN in this case, but a pathological condition might be related to the lectin pathway, in addition to the alternative pathway. Karashima et al. observed predominant IgA deposition on the glomerular capillary wall and IgA2 deposition in two cases of secondary MPGN caused by a congenital portosystemic shunt [8]. In those two cases, hyperammonemia, elevated bile acids, and symptoms associated with hyperammonemia were observed from one to several years after the occurrence of urinary protein. In our case, a congenital portosystemic shunt was not detected by abdominal ultrasonography, computed tomography, and blood tests.

Growth hormone (GH) treatment was initiated when the patient was 4 years and 5 months of age due to her short stature. Four cases of IgA nephropathy during GH treatment have been reported in Japan. All of these cases were undergoing current GH treatment. One of these patients had stopped GH treatment because a renal biopsy had shown diffuse mesangial proliferation with cellular crescents in more than 30% of the glomeruli [9–12]. In our case, GH therapy was completed 1 year and 2 months before the onset of MPGN, so the effect of GH therapy is considered to be low.

MPGN is a progressive primary glomerulonephritis that occurs primarily in children and young adults [13]. Many studies have demonstrated that pediatric MPGN patients with alternate-day prednisolone treatment have a good prognosis [14–17]. In addition, treatment options, including angiotensin-converting enzyme inhibitors, angiotensin II receptor blockers, immunosuppressive agents, plasma exchange, and anti-inflammatory agents, could be administered depending on the amount of urinary protein, renal impairment, and steroid response [18]. However, to date, a consensus has not yet been reached regarding the administration method and the therapeutic effect of steroids in MPGN patients, and few studies have examined CsA use in MPGN cases [19, 20]. In our case, after three courses of steroid pulse therapy and oral administration of prednisolone, the urinary protein levels did not decrease, and lisinopril and MZR were thus added. However, her urinary protein tests did not become negative, and hematuria persisted. Hence, we added two courses of steroid pulse therapy, and the immunosuppressive drug was changed from MZR to CsA. Finally, the patient was in remission. These findings suggest that CsA may be useful as an immunosuppressive agent in patients with refractory MPGN.

In conclusion, we report a case of MPGN with IgA deposits along the glomerular capillary wall and in the mesangial region. The treatment for this case was difficult and complicated, and the CsA treatment resulted in remission. Our findings suggest that CsA may be useful as an immunosuppressant against refractory MPGN.

## Data Availability

All data generated or analyzed during this study are included in this published article.

## Abbreviations

MPGN: Membranoproliferative glomerulonephritis
MPT: Methylprednisolone pulse therapy
CsA: Cyclosporine
SD: Standard deviation
MZR: Mizoribine
GH: Growth hormone
